# The effects of an 8-week complex training program on lower limb explosive power and movement agility in adolescent female badminton players

**DOI:** 10.3389/fphys.2025.1678866

**Published:** 2025-09-22

**Authors:** Rui Hu, Minchen Zhao, Xiang Shen, Zhiming Wang

**Affiliations:** ^1^ Graduate Department, Nanjing Sport Institute, Nanjing, China; ^2^ School of Physical Education, Guangzhou College of Commerce, Guangzhou, China; ^3^ School of Sports Training, Nanjing Sport Institute, Nanjing, China

**Keywords:** complex training, adolescent female athletes, badminton, explosivepower, agility, post-activation performance enhancement

## Abstract

**Background:**

Badminton requires high-intensity lower limb explosive power and agility for executing rapid lunges, jumps, and directional changes. Complex training (CT) combines heavy resistance exercises with plyometric activities to enhance maximal strength and explosive performance through post-activation performance enhancement (PAPE). However, limited research exists on CT effects in adolescent female badminton players.

**Objective:**

To investigate the effects of an 8-week complex training program on lower limb explosive power and movement agility in adolescent female badminton players.

**Methods:**

Thirty-two adolescent female badminton players were randomly allocated into complex training group (CT group, n = 16, age: 15.69 ± 0.95 years) and resistance training group (RT group, n = 16, age: 15.63 ± 1.15 years). The CT group performed resistance exercises (75%–85% 1RM) paired with plyometric exercises twice weekly for 8 weeks, while the RT group completed traditional resistance training with the same frequency. Pre- and post-intervention assessments included squat jump (SJ), countermovement jump (CMJ), bilateral and unilateral jumps, sprint tests (5 m, 10 m, 15 m, 20 m), hexagon test, modified 505 change of direction (COD) test, on-court COD test, and isometric mid-thigh pull (IMTP). Two-way repeated measures ANOVA was used for statistical analysis (p < 0.05).

**Results:**

Significant group × time interactions were observed for SJ, CMJ, unilateral jumps, sprint performance (5 m, 10 m, 20 m), and agility tests (all p < 0.05). The CT group showed greater improvements compared to RT group: SJ (1.83 cm vs. 0.95 cm, Cohen’s d = 1.196 vs. 0.642), CMJ (3.64 cm vs. 1.27 cm, Cohen’s d = 1.949 vs. 0.681), 5 m sprint (0.18s vs. 0.06s improvement, Cohen’s d = 1.889 vs. 0.667), hexagon test (1.29s vs. 0.03s improvement), and COD performance. Both groups significantly improved IMTP with no between-group differences (p > 0.05).

**Conclusion:**

An 8-week complex training program elicited significantly greater improvements in lower limb explosive power, sprint acceleration, and multidirectional agility compared to traditional resistance training in adolescent female badminton players. These findings suggest CT is an effective, sport-specific training intervention that capitalizes on the heightened neuromuscular plasticity of adolescent athletes.

## Introduction

Agility and high-intensity bursts of force in the lower limbs are essential in badminton. Players often execute explosive lunges, jumps, and swift changes of direction in response to the shuttlecock’s unpredictable trajectory (Sonoda et al., 2018). These complex movements heavily rely on lower-limb explosive power and agility, particularly for adolescent female players, influences on-court performance and potentially reduces injury risks by enhancing neuromuscular control (Gaamouri et al., 2023). Specific training is crucial in badminton due to its unique movement patterns, including rapid lateral shuffles, multidirectional lunges, and intermittent jumps, which place significant demands on the lower-limb muscles (Lu et al., 2025). Traditional resistance training methods, although effective in enhancing maximal strength, often lack the necessary movement specificity to translate to the explosive, multidirectional requirements of badminton.

Complex training (CT) is a method that combines heavy resistance exercises with plyometric activities in a single session to improve maximal strength and explosive performance (Wang et al., 2023). Implementing a structured CT protocol that integrates resistance exercises like back squats with plyometric exercises such as drop jumps and box jumps is beneficial for enhancing the movement specificity required for explosive, multidirectional performance in sports like badminton. This training approach capitalizes on post-activation performance enhancement (PAPE), temporarily boosting subsequent explosive output by increasing motor unit recruitment and enhancing muscle fiber synchronization through high-intensity contractions (Ciocca et al., 2021). By targeting the efficiency of the stretch–shortening cycle (SSC), CT has the potential to enhance powerful and rapid movements (Turner and Jeffreys, 2010). Unlike isolated resistance training, which focuses on maximal force production, or plyometric training, which emphasizes reactive strength (Allégue et al., 2023), CT aims to combine the advantages of both methods to enhance dynamic movements essential for sports like badminton.

Several meta-analyses and systematic reviews have advanced our understanding of CT. Bauer et al. (2019) demonstrated that integrating both higher- and lower-load exercises in a single session effectively enhances key performance indicators such as countermovement jump height, squat jump performance, and sprint times. Similarly, Freitas et al. (2017) showed that CT interventions result in moderate to substantial improvements in sprint performance and vertical jump ability among team-sport athletes. These findings are consistent with research in various sports, including basketball and rugby (Freitas et al., 2019; Comyns et al., 2010). Recent investigations have also begun to explore CT in racket sports, such as its application to improve biomechanical characteristics of lower limbs during badminton-specific movements like the backhand forward lunge (Xie et al., 2025). Despite the robust evidence supporting the effectiveness of complex training, research has predominantly focused on male athletes or mixed cohorts, creating a noticeable gap in the literature regarding adolescent female badminton players. This gap is particularly significant given the sport’s demands for rapid, multidirectional movements that require high levels of lower-limb explosive power and agility, which may interact uniquely with the physiological and neuromuscular changes occurring during female adolescence. For instance, adolescent females experience rapid growth spurts, hormonal fluctuations (e.g., estrogen surges), and shifts in body composition that can affect muscle strength, joint stability, and neuromuscular coordination (Reich et al., 2023). These changes may heighten vulnerability to injuries, such as anterior cruciate ligament strains common in multidirectional sports (Zebis et al., 2022), while also presenting a window for targeted training to optimize performance adaptations (Van Hooren and De Ste Croix, 2020). In badminton, where players must frequently perform explosive lunges and quick directional changes under fatigue, these developmental factors could amplify the need for specialized interventions like CT to improve force production efficiency and movement specificity. Addressing this population is thus essential not only for scientific advancement—by extending CT evidence to underrepresented groups—but also for practical applications, such as designing training programs that enhance on-court agility, reduce injury risks through better neuromuscular control, and support long-term athlete development in a sport with growing female participation.

Adolescent female athletes constitute a distinctive group within the athletic community due to the rapid physiological and neuromuscular advancements and hormonal fluctuations they encounter during adolescence (Reich et al., 2023). These factors significantly influence muscle strength, coordination, and overall athletic performance. Particularly in female athletes, these transformations can notably impact movement mechanics and the likelihood of sustaining injuries. Consequently, it is imperative that training programs tailored to this demographic are meticulously devised to foster development while minimizing potential risks. Despite existing research on the effects of resistance and plyometric training on performance in adolescents (Santos and Janeira, 2008), limited attention has been given to adolescent female badminton players. This oversight is critical, as CT could offer substantial practical benefits for this group, including improved explosive power for better shot execution and agility for superior court coverage, ultimately leading to enhanced competitive performance. Moreover, by strengthening lower-limb muscles and refining SSC efficiency, CT may help mitigate common injuries like ankle sprains or knee issues prevalent in badminton, thereby promoting safer training environments and sustained athletic progression during a pivotal developmental stage. Given the sport’s unique movement characteristics and the developmental phase of these athletes, a focused investigation is crucial for gaining insights into how intricate training interventions could enhance on-court performance.

Therefore, the aim of this study was to explore the effects of an 8-week complex training program on lower limb explosive power and movement agility in adolescent female badminton players. The hypothesis was that an 8-week complex training can significantly improve the lower limb explosive power and movement agility in adolescent female badminton players.

## Methods

### Experimental approach to the problem

**FIGURE 1:**
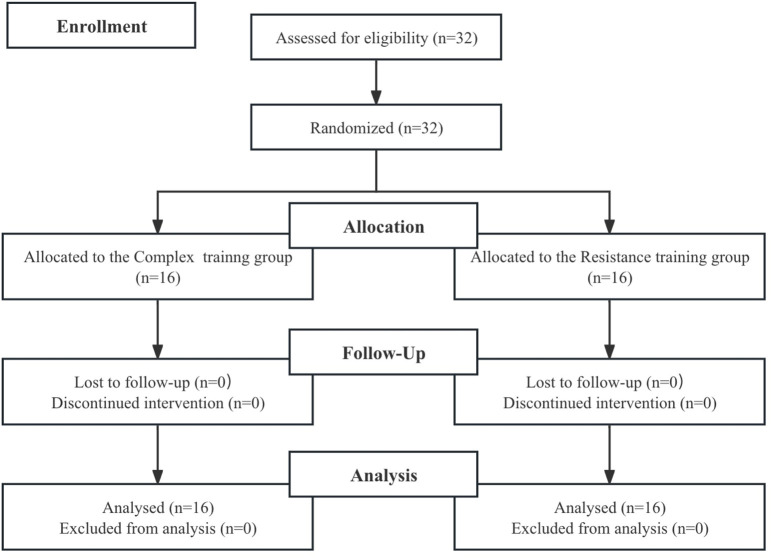
Flow chart of the progress through the phases of the study according to the CONSORT statements.

This study adopted a randomized controlled trial design involving 32 adolescent female badminton players (Figure 1). Participants were randomly assigned to either a complex training group (CT, n = 16; age: 15.69 ± 0.95 years; height: 166.64 ± 6.16 cm; body mass: 53.98 ± 2.91 kg) or a resistance training group (RT, n = 16; age: 15.63 ± 1.15 years; height: 162.95 ± 5.83 cm; body mass: 54.65 ± 4.25 kg). All participants were national- or regional-level athletes with at least 6 years of systematic badminton training and an average weekly training volume of approximately 18 h. None of the participants reported musculoskeletal injuries within the previous year.

We conducted *post hoc* power analyses based on the effect sizes estimated from the study’s own data rather than assumed values. Specifically, we first identified the lowest and highest partial eta-squared (ηp^2^) among the significant group × time interactions reported in the Results and converted them to Cohen’s f using

$$f=\sqrt{\eta_{p}^{2}}\left(1-\eta_{p}^{2}\right)$$

The lowest ηp^2^ was observed for the 20 m sprint (ηp^2^ = 0.182; *f* = 0.472), whereas the highest ηp^2^ was observed for the unilateral countermovement jump on the non-dominant side (CMJ-ND; ηp^2^ = 0.554; *f* = 1.115). Using G*Power (version 3.1; test family: *F* tests; ANOVA: repeated measures, within–between interaction; α = 0.05; total sample size = 32; number of groups = 2; measurements = 2; correlation among repeated measures = 0.5; ε = 1), the achieved power (1–β) was computed for these two effect sizes and reported in the Results as a range to transparently reflect statistical adequacy across different effect magnitudes.

The study was approved by the Institutional Ethics Committee of Guangzhou College of Commerce (Approval No. 2024075) and conducted in accordance with the Declaration of Helsinki. Written informed consent was obtained from all participants and their legal guardians.

### Measures

To minimize potential learning effects, all participants completed a familiarization session 1 week prior to baseline testing. During this session, athletes were introduced to all testing procedures and performed supervised practice trials. For the jump assessments (squat jump, bilateral and unilateral countermovement jumps), participants executed 2–3 submaximal attempts to refine technique, followed by 1–2 maximal practice trials, with only the best effort verbally reinforced and no data recorded. For the linear sprint tests over 5, 10, 15, and 20 m, athletes performed two submaximal accelerations at approximately 70%–80% of perceived maximum effort and one full-speed trial to become accustomed to the electronic timing system. The agility assessments, including the hexagon agility test, the modified 505 COD test, and the on-court COD test, each involved 1–2 submaximal familiarization runs and one maximal effort attempt. For the isometric mid-thigh pull, participants practiced posture and pulling technique with the bar fixed, followed by two submaximal pulls (∼50–70% effort) and one maximal attempt. The familiarization session lasted approximately 60–75 min, and no performance data were retained for analysis. This procedure ensured that all participants were adequately accustomed to the protocols and helped minimize potential learning effects during formal testing, which was subsequently conducted across three non-consecutive days separated by 24 h (Day 1: jump and sprint tests; Day 2: agility tests; Day 3: IMTP). To control for potential fatigue effects associated with administering multiple tests on the same day, all assessments were conducted in a standardized sequence with prescribed rest intervals. On Day 1, participants first completed the SJ, followed by the bilateral and then unilateral CMJ, before performing the linear sprint tests over 5, 10, 15, and 20 m. On Day 2, the testing order was the hexagon agility test, the modified 505 COD test, and the on-court COD test. Day 3 was reserved exclusively for the IMTP. To minimize carry-over fatigue, athletes rested for approximately 3 min between repeated attempts of the same test and 5 min between different tests. These intervals allowed adequate recovery while maintaining session efficiency. Moreover, verbal encouragement and consistent supervision ensured that each test was performed with maximal effort. This procedure was designed to reduce the potential impact of fatigue on performance outcomes and to enhance the reliability and reproducibility of the results. For each jump and sprint assessment, participants performed three maximal trials, with the best performance retained for analysis. Rest intervals of approximately 2–3 min were provided between repeated attempts of the same test to minimize fatigue and ensure recovery. For the IMTP, participants completed three maximal pulls, each separated by a 3-min rest interval, and the highest peak force value was used for analysis. Similarly, for the agility assessments, two maximal trials were performed with 3 min of rest between trials, and the better performance was recorded. This approach was chosen to reduce potential fatigue effects and to enhance the reliability of the measurements.

To minimize confounding factors, participants were instructed to avoid any strenuous physical activity for at least 24 h prior to each testing session and to maintain a fasting state for at least 2 hours before assessments. Before each testing session, a standardized 8–10 min warm-up was performed, consisting of jump rope exercises, general dynamic mobility drills, multidirectional acceleration runs, and progressively intensified jumping movements.

#### Countermovement jump (CMJ) test

Bilateral and unilateral (e.g., dominant and non-dominant side) CMJ without an arm swing were performed on an infrared plate Optojump (Microgate, Bolzano, Italy), according to procedures previously described (Madruga-Parera et al., 2020). During each trial, participants began from an upright standing position with hands placed on the hips. They then executed a rapid downward movement to a self-selected depth, followed by an immediate vertical jump performed with maximal effort.

#### Squat jump test (SJ)

For the SJ, players were instructed to hold a static squat position with 90° knee flexion for 3 s before jumping. The jump height was calculated from the flight time data derived from a jump mat an infrared plate Optojump (Microgate, Bolzano, Italy).

#### Pre-stretch augmentation percentage

PSAP was used to indirectly examine the ability of an athlete to use the stretch-shortening cycle (SSC) to improve their jump height and peak power during a vertical jump, which was often used as an indicator of lower-limb power performance (Suchomel et al., 2016). Indices from the jump data were PSAP and were calculated as follows:

$$PSAP=\frac{CMJheight-SJheight}{SJheight}\times 100\%$$

#### Hexagon test

The hexagon test was used to assess agility and coordination. Participants stood facing forward at the center of a hexagon marked on the floor, with each side measuring 60 cm and internal angles of 120°. With feet together and hips aligned forward, participants performed consecutive two-footed hops over each side of the hexagon in a clockwise direction, completing three full sequences as quickly as possible (Beekhuizen et al., 2009). Performance was recorded using an iPhone XS (Apple Inc., Cupertino, CA, United States) running iOS 13.7, mounted on a GripTight Mount Pro tripod (Joby, United States) positioned 1 m from the testing area. All trials were captured at 240 Hz and analyzed using Kinovea software (version 0.8.15; available at http://www.kinovea.org). Time penalties were applied to account for execution errors: 0.5 s were added for stepping on a line and 1.0 s for deviating from the prescribed sequence. Each participant was allowed one familiarization trial before performing three official attempts, with a 45-s passive rest between trials. The shortest completion time across the three trials was used for further analysis (Beekhuizen et al., 2009).

#### Sprint test

Sprint performance over 20 m, including 5 m, 10 m, 15 m, and 20 m split times, was assessed using single-beam photocell timing gates (Microgate, Bolzano, Italy) positioned 1.0 m above ground level. Each sprint was initiated from a standing start, with the participant positioned 50 cm behind the first timing gate to ensure consistent trigger activation.

#### Modified 505 COD test

The abilities of players to perform a single, rapid 180° change of direction over a 5 m distance was measured using a modified version (stationary start) of the 505 COD test (Fernandez-Fernandez et al., 2016). Players started in a standing position with their preferred foot 0.5 m behind the starting line. They were asked to plant their preferred (i.e., considered as the dominant side) foot on executing the turn. Three trials were completed, and the best time was recorded (Microgate, Bolzano, Italy). Two minutes of rest were allowed between trials. The COD_DEF_ for the 505 test was calculated using the following formula: COD_DEF_=(modified 505 time–10 m time) (Nimphius et al., 2013).

#### On-court COD test

**FIGURE 2:**
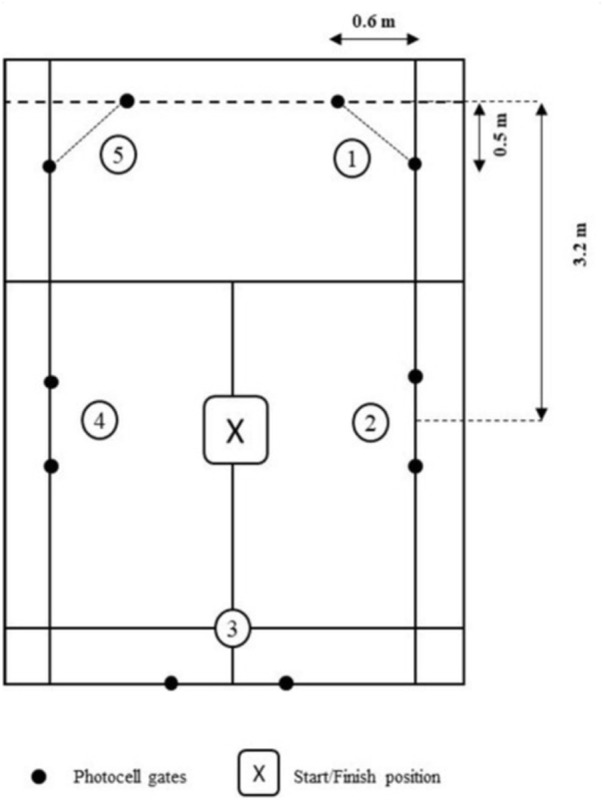
On-Court COD test.

Players performed an adapted version of a previously published footwork testAn adapted version of the badminton-specific footwork agility test originally developed by Chin et al. (1995) was used to evaluate on-court change of direction (COD) performance. The test was conducted on one-half of a standard badminton court. Five pairs of photocell timing gates (Microgate, Bolzano, Italy) were mounted at two heights (0.5 m and 1.0 m) to detect athlete movement, as illustrated in Figure 2.

Participants began at the central point of the court and were instructed to sprint as quickly as possible toward the first target location (forecourt right side, #1 in Figure 2), cross through the designated photocell gate with their waist (verified visually by the researchers), and immediately return to the center. They then proceeded to the next target location in a predefined sequence (#2 through #5), using badminton-specific movement patterns such as lateral shuffles, crossover steps, and forward lunges. The test concluded once the player returned to the central point after completing all five directional movements.

#### Isometric mid-thigh pull test

The isometric mid-thigh pull (IMTP) test was employed as a safe, reliable, and time-efficient method to assess lower limb maximal strength in adolescent athletes, as recommended by Till et al. (2018). Prior to testing, the mid-thigh position for each participant was identified by marking the midpoint between the knee and hip joints. Participants then assumed their preferred deadlift stance by self-selecting hip and knee joint angles, ensuring a position that closely mimicked their typical lifting posture.

The barbell height was adjusted accordingly to align with the marked mid-thigh position. Participants were permitted to use either an overhand, mixed, or hook grip. Upon instruction, they were required to exert maximal force by pulling upward on the barbell as quickly and forcefully as possible and to sustain their effort for 6 s. To minimize anticipatory muscle activation, participants were instructed to remain relaxed until the verbal cue “GO” was given.

Ground reaction forces during each trial were recorded using a force plate (Kistler 9281CA, Kistler, Winterthur, Switzerland) at a sampling frequency of 1,000 Hz, following the protocol outlined by Comfort et al. (2015). Peak force was defined as the maximum force output during the 6-s trial, adjusted by subtracting the participant’s body weight (in Newtons). Additionally, force values at specific time intervals (30, 50, 90, 100, 150, 200, and 250 ms) from the initiation of force application were extracted for further analysis.

### Training program

In the CT group, participants trained 2 days per week for a period of 8 weeks (Mondays and Fridays), three stages each session lasted 60 min, training sessions were interspersed by 48–72 h. For novice (untrained individuals with no RT experience or who have not trained for several years) training, resistance training using progressive resistance training protocols (American College of Sports Medicine position stand, 2009). Selection of training movements according to the sports of the badminton movement pattern, five pairs of training movements, each consisting of one resistance training exercise and one plyometric exercise, select 3 sets of movements for each training session. Within each complex pair, a 3 min rest interval was allowed because a similar interval had been demonstrated to elicit an optimal PAPE effect (Mina et al., 2019). In addition, a 4 min rest interval was included between complex pairs since 2–5 min interest recovery is suggested to produce the greatest strength benefits (Suchomel et al., 2018). Participants in the RT group completed a 8-week RT intervention. The RT was completed on the same day as CT. On each training day, participants were asked to complete resistance training movements (Table 1, 2). One-repetition maximum (1RM) test for each movement was assessed once every 2 weeks to adjust the training plan. The 1RM for all resistance exercises was assessed every 2 weeks to adjust training loads. To minimize excessive fatigue, 1RM values were not determined through exhaustive direct testing on a single maximal attempt. Instead, they were estimated using the multiple-repetition maximum method, in which participants performed submaximal lifts (typically 3–5 repetitions to failure), and 1RM was subsequently calculated using validated prediction equations. This approach has been shown to provide reliable estimates of maximal strength while reducing the fatigue and injury risk associated with traditional direct 1RM testing.

**TABLE 1:** Complex training program protocol performed by the complex-paired training group.

Training schedule		Complex pair	Intensity			Sets* repetitions	Rest (min)
			The first stage (1–2 weeks)	The second stage (3–5 weeks)	The third stage (5–8 weeks)		
The first and second weeks	Monday	Squat + Squat Jump	75%1RM + ME	80%1RM + ME	85%1RM + ME	3*(4∼6 + 10∼12)	3 min
		Barbell bench press + High-five push-ups	75%1RM + ME	80%1RM + ME	85%1RM + ME	3*(4∼6 + 10∼12)	3 min
	Friday	Deadlift + High pull	75%1RM+50%1RM	80%1RM+50%1RM	85%1RM+50%1RM	3*(4∼6 + 10∼12)	3 min
		Loaded pull-ups + Elastic band pull-down	75%1RM + ME	80%1RM + ME	85%1RM + ME	3*(4∼6 + 10∼12)	3 min
The third and fourth weeks	Monday	Weight-bearing lunge + Split-leg squat jump	75%1RM + ME	80%1RM + ME	85%1RM + ME	3*(4∼6 + 10∼12)	3 min
		Dumbbell bench press + Kneeling forward medicine ball	75%1RM + ME	80%1RM + ME	85%1RM + ME	3*(4∼6 + 10∼12)	3 min
	Friday	Military press + Push press	75%1RM+50%1RM	80%1RM+50%1RM	85%1RM+50%1RM	3*(4∼6 + 10∼12)	3 min
		Reverse grip loaded pull-ups + Elastic band pull-up	75%1RM + ME	80%1RM + ME	85%1RM + ME	3*(4∼6 + 10∼12)	3 min

1RM, 1-repetition maximum; ME, maximal effort.

Loaded pull-ups, use the belt to hang the barbell plate to increase the load; Elastic band pull-down and Elastic band pull-up, the first stage uses about 35 pounds of the elastic band, and the second and third stages add 5 pounds to the previous stage; Weight-bearing lunge, use dumbbells to increase the load, alternating feet; Kneeling forward medicine ball, facing the wall, throw the medicine ball forward with both hands, the first stage uses an 8 kg medicine ball, and the second and third stages add 2 kg to the previous stage.

**TABLE 2:** Resistance-training program protocol performed by the resistance training group.

Training schedule		Resistance-training	Intensity			Sets* repetitions	Rest (min)
			The first stage (1–2 weeks)	The second stage (3–5 weeks)	The third stage (5–8 weeks)		
The first and second weeks	Monday	Squat	75%1RM	80%1RM	85%1RM	6*(6∼10)	3 min
		Barbell bench press	75%1RM	80%1RM	85%1RM	6*(6∼10)	3 min
	Friday	Deadlift	75%1RM	80%1RM	85%1RM	6*(6∼10)	3 min
		Loaded pull-ups	75%1RM	80%1RM	85%1RM	6*(6∼10)	3 min
The third and fourth weeks	Monday	Weight-bearing lunge	75%1RM	80%1RM	85%1RM	6*(6∼10)	3 min
		Dumbbell Bench Press	75%1RM	80%1RM	85%1RM	6*(6∼10)	3 min
	Friday	Military press	75%1RM	80%1RM	85%1RM	6*(6∼10)	3 min
		Loaded pull-ups	75%1RM	80%1RM	85%1RM	6*(6∼10)	3 min

1RM, 1-repetition maximum; ME: maximal effort.

### Statistical analysis

All experimental data were processed using JASP (version 0.18.3, JASP team, Netherlands) and expressed as mean ± standard deviation (M ± SD). The normality of data distribution was assessed using the Shapiro-Wilk test. Baseline differences between groups were examined using one-way ANOVA for normally distributed data. Sphericity assumption was assessed using Mauchly’s test. If the sphericity assumption was violated and Epsilon (ε) < 0.75, the Greenhouse–Geisser correction was applied. If ε > 0.75, the Huynh–Feldt correction was used. A two-way repeated measures ANOVA was conducted to examine the training effects, with group (CT and RT) and time (pre- and post-intervention) as factors, and their interaction. When significant interactions were observed, Bonferroni post-hoc comparisons were performed to identify specific differences. Effect sizes were calculated using Cohen’s d and classified as trivial (d < 0.2), small (0.2 ≤ d ≤ 0.6), moderate (0.6 ≤ d ≤ 1.2), large (1.2 ≤ d ≤ 2.0), or very large (d > 2.0) (Cohen, 2013). The significance level was set at p < 0.05 for all analyses.

## Results

All the participants completed this study, and all the data were included in the analysis. No significant difference in the outcomes measured were observed between both group (P > 0.233). Based on the lowest partial eta-squared value (20 m sprint; ηp^2^ = 0.182, f = 0.472), the *post hoc* statistical power of the present design was 0.73. Based on the highest partial eta-squared value (CMJ-ND; ηp^2^ = 0.554, f = 1.115), the *post hoc* statistical power was 1.00.

**FIGURE 3:**
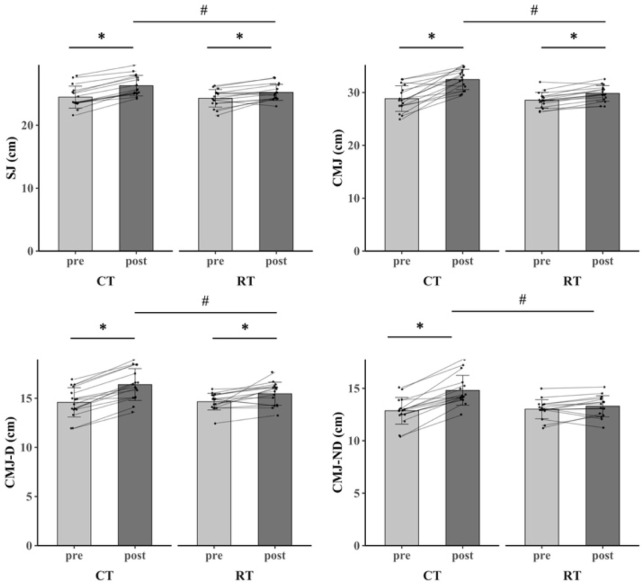
The jump outcomes for CT group and RT group before and after 8-week training.

**FIGURE 4:**
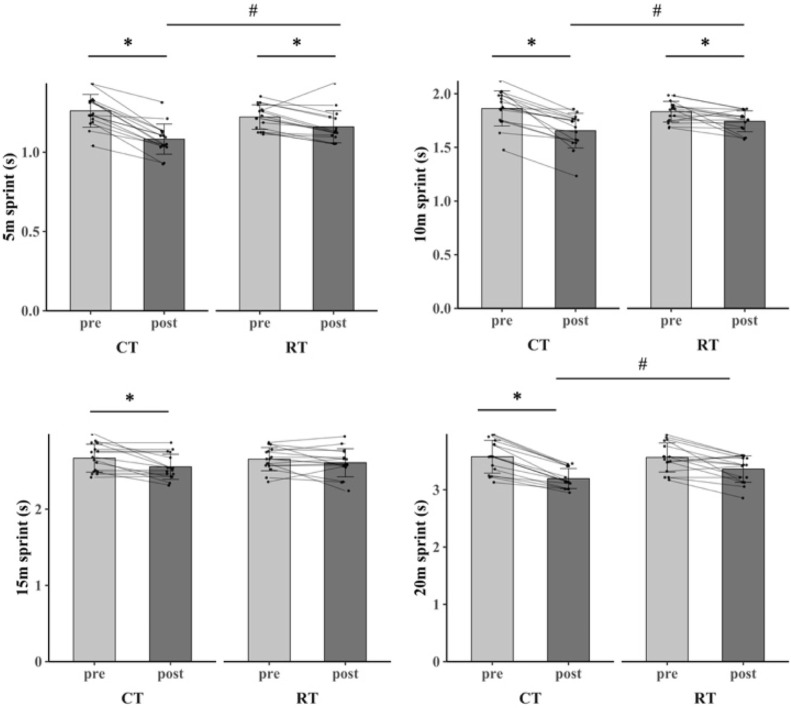
The sprint outcomes for CT group and RT group before and after 8-week training.

**FIGURE 5:**
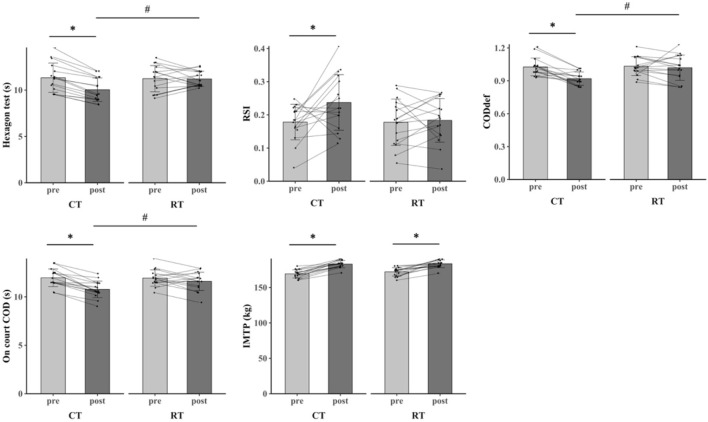
The change of direction outcomes for CT group and RT group before and after 8-week training.

The primary two-way repeated-measures ANOVA models showed significant main effects of time, and interactions between group and time on SJ (ηp^2^ = 0.248, %Δ = 7.48%), CMJ (ηp^2^ = 0.360, %Δ = 12.63%), CMJ-D (ηp^2^ = 0.257, %Δ = 12.41%), CMJ-ND (ηp^2^ = 0.554, %Δ = 15.25%), 5 m sprint (ηp^2^ = 0.369, %Δ = 14.29%), 10 m sprint (ηp^2^ = 0.189, %Δ = 10.75%), 20 m sprint (ηp^2^ = 0.182, %Δ = 10.89%), hexagon test (partial ηp^2^ = 0.333, %Δ = 11.39%), COD_DEF_ (ηp^2^ = 0.220, %Δ = 10.68%), and on court COD (ηp^2^ = 0.245, %Δ = 9.95%). The post-hoc analysis revealed that SJ, CMJ, CMJ-D, CMJ-ND, 5 m sprint, 10 m sprint, 20 m sprint, hexagon test, COD_DEF_, and on court COD in CT group were significantly greater after the intervention compared to all the other pre- and post-interventions (P < 0.001). Within RT group, SJ (P < 0.001), CMJ (P = 0.024), CMJ-D (P = 0.006), 5 m sprint (P = 0.018), 10 m sprint (P = 0.035), and 20 m sprint (P < 0.001) were significantly greater after training as compared to baseline (Table 3; Figures 3–5).

**TABLE 3:** The outcomes for CT group and RT group before and after 8-week training.

Variable	CT (N = 16)			RT (N = 16)			P-value		
	Pre	Post	Cohen’s d (%Δ)	Pre	Post	Cohen’s d (%Δ)	Time	Group	Interaction
SJ (cm)	24.47 ± 1.76	26.30 ± 1.63*#	1.196 (7.48%)	24.28 ± 1.40	25.23 ± 1.28*	0.642 (3.91%)	<0.001	0.240	0.004
CMJ (cm)	28.83 ± 2.42	32.47 ± 1.92*#	1.949 (12.63%)	28.55 ± 1.48	29.82 ± 1.47*	0.681 (4.45%)	<0.001	0.019	<0.001
CMJ-D (cm)	14.58 ± 1.49	16.39 ± 1.62*#	1.369 (12.41%)	14.65 ± 0.85	15.46 ± 1.18*	0.611 (5.53%)	<0.001	0.332	0.003
CMJ-ND (cm)	12.85 ± 1.28	14.81 ± 1.43*#	1.660 (15.25%)	13.01 ± 0.91	13.29 ± 1.01	0.704 (2.15%)	<0.001	0.092	<0.001
5 m sprint (s)	1.26 ± 0.10	1.08 ± 0.10*#	1.889 (14.29%)	1.22 ± 0.08	1.16 ± 0.10*	0.667 (4.92%)	<0.001	0.510	<0.001
10 m sprint (s)	1.86 ± 0.16	1.66 ± 0.16*#	1.533 (10.75%)	1.83 ± 0.10	1.74 ± 0.10*	0.678 (4.92%)	<0.001	0.013	0.013
15 m sprint (s)	2.67 ± 0.18	2.56 ± 0.17*	0.657 (4.12%)	2.66 ± 0.15	2.61 ± 0.18	0.287 (1.88%)	0.004	0.710	0.231
20 m sprint (s)	3.58 ± 0.28	3.19 ± 0.18*#	1.588 (10.89%)	3.56 ± 0.26	3.36 ± 0.23*	0.851 (5.62%)	<0.001	0.332	0.015
Hexagon test (s)	11.33 ± 1.59	10.04 ± 1.27*#	0.990 (11.39%)	11.23 ± 1.41	11.20 ± 0.83	0.025 (0.27%)	<0.001	0.224	<0.001
RSI	0.18 ± 0.05	0.24 ± 0.09*	0.854 (33.33%)	0.17 ± 0.07	0.18 ± 0.06	0.090 (5.88%)	0.026	0.199	0.067
COD_DEF_	1.03 ± 0.08	0.92 ± 0.06*#	1.247 (10.68%)	1.03 ± 0.09	1.02 ± 0.12	0.164 (0.97%)	<0.001	0.053	0.007
On court COD (s)	11.96 ± 0.92	10.77 ± 0.85*#	1.341 (9.95%)	11.93 ± 0.84	11.60 ± 0.95	0.381 (2.77%)	<0.001	0.170	0.004
IMTP (kg)	169.19 ± 5.86	182.91 ± 5.19*	2.430 (8.11%)	172.08 ± 6.00	183.52 ± 5.50*	2.025 (6.65%)	<0.001	0.286	0.339

*: significant difference between pre- and post-intervention.

#: significant difference between CT group and RT group.

The primary two-way repeated-measures ANOVA models showed significant main effects of time but no interactions between group and time on 15 m sprint, RSI, and IMTP. Within CT group, 15 m sprint (P = 0.027), RSI (P = 0.032), and IMTP (P < 0.001) were significantly greater after training as compared to baseline.

## Discussion

The primary aim of this study was to explore whether an 8-week complex training program could enhance lower limb explosive power and movement agility in adolescent female badminton players. The major findings demonstrated that the CT group achieved significantly greater improvements in SJ, CMJ, sprint performance (5 m, 10 m, 20 m), COD ability, and RSI, when compared to a traditional resistance training group. Notably, agility-related performance and unilateral jump ability exhibited particularly pronounced gains in the CT group.

The present results align with a substantial body of literature indicating that CT can effectively improve explosive power and agility across various sports contexts in young athletes (Gee et al., 2021; Beato et al., 2018; Cavaco et al., 2014; Oliver et al., 2024). Similar to findings in male youth soccer players (Oliver et al., 2024), adolescent female badminton players in this study experienced moderate-to-large improvements in jump performance and acceleration. Importantly, our study extends these observations to young female athletes engaged in a sport that uniquely combines frequent unilateral explosive movements and high agility demands.

In terms of jump performance, improvements in both bilateral and unilateral CMJ and SJ were found in the CT group. These findings are consistent with prior studies reporting CT-induced gains in vertical and horizontal power among team sport athletes (Wang et al., 2024; Freitas et al., 2017). However, few previous studies have examined unilateral performance in badminton players, especially in adolescent female athletes. Unilateral strength plays a crucial role in badminton due to its inherently asymmetrical movement patterns and load distribution (Cronin et al., 2003). In our study, both CMJ-D and CMJ-ND showed large effect sizes (d = 1.369 and 1.660, respectively), suggesting that this group may respond particularly well to CT, potentially due to the high neuromuscular plasticity associated with their developmental stage (Oliver et al., 2024; Chatzinikolaou et al., 2018) and the need for asymmetrical strength adaptations in badminton. Moreover, the sport-specific integration of unilateral plyometric drills may have enhanced inter-limb coordination and stretch-shortening cycle efficiency more effectively than traditional RT alone.

Regarding sprint performance, the CT group exhibited significantly greater improvements in 5 m, 10 m, and 20 m sprint times compared to the RT group, as indicated by significant group-by-time interaction effects. This is consistent with existing CT literature emphasizing the role of post-activation performance enhancement (PAPE) in facilitating initial acceleration (Xu et al., 2025). For adolescent female badminton players, who must frequently execute short bursts of acceleration rather than sustained sprints, these gains are highly relevant. Interestingly, the improvements in 15 m sprint and RSI were significant but not accompanied by group-time interaction effects. The corresponding effect sizes (d = 0.657 for 15 m sprint and 0.854 for RSI) were moderate, suggesting that while CT is beneficial for these metrics, further tailoring of sprint and plyometric content may be required to optimize performance gains beyond the initial acceleration phase.

The most pronounced benefits of CT were observed in COD and agility performance, as evidenced by superior improvements in the hexagon test, modified 505 COD, and on-court COD outcomes. These improvements were not only statistically significant but also accompanied by large effect sizes (e.g., d = 0.990 for the hexagon test, d = 1.247 for CODDEF, and d = 1.341 for on-court COD), indicating substantial practical enhancements in multidirectional movement ability. In contrast, the RT group exhibited minimal agility improvements, with negligible or small effect sizes. This finding aligns with prior research highlighting that CT may offer greater movement specificity and transfer to agility tasks (Forster et al., 2022). For adolescent female athletes, this is particularly valuable, as this demographic often experiences neuromuscular control deficits and increased injury risk during pubertal growth (Bencke et al., 2018). Our findings suggest that CT may not only enhance agility but also contribute to movement stability and injury prevention, addressing critical developmental concerns for young female badminton players.

Finally, although both groups improved isometric strength (IMTP), no significant group-time interactions were observed. Both the CT and RT groups exhibited large effect sizes for IMTP (CT: d = 2.430; RT: d = 2.025), indicating substantial within-group improvements. Notably, the effect size was larger in the CT group, suggesting a potentially greater neuromuscular adaptation, even though the between-group difference was not statistically significant. This finding is consistent with Oliver et al. (2024), who reported that maximal isometric strength gains do not necessarily translate to dynamic performance improvements unless accompanied by movement-specific adaptations. In this context, the superior agility and jump outcomes observed in the CT group may be attributed more to improved neuromuscular coordination and stretch-shortening cycle utilization than to increases in maximal strength alone.

Although the present findings confirm that CT leads to greater improvements in explosive power and agility indicators such as CMJ, 5 m sprint, and COD performance, it is important to acknowledge that some studies report comparable or even limited effects of CT compared to traditional resistance training (RT). For example, Miranda et al. (2022) found no significant differences between CT and RT across various performance indicators, including SJ, CMJ, CODS, and sprinting ability in young soccer players. Similarly, Schneiker et al. (2023) and Rathi et al. (2023)observed no meaningful advantage of CT over RT in strength, jumping, and sprint-based assessments across diverse athletic populations. In the current meta-analysis (Thapa et al., 2024), CT and RT showed equivalent outcomes in 1RM squat, SJ, RSI, and longer sprint distances (10–60 m), suggesting that the benefits of CT may be task-and context-specific. Furthermore, the certainty of the evidence was rated from low to very low, underscoring the need for cautious interpretation. These contrasting findings imply that while CT offers performance gains in specific parameters-especially in explosive short-distance movements-its superiority over RT is not universal. Incorporating such varied evidence highlights the nuanced efficacy of CT and supports a more balanced interpretation of its utility in athletic training programs.

Several limitations should be acknowledged when interpreting the present findings. First, although all participants were high-level adolescent female badminton players with comparable training backgrounds, individual differences in biological maturation were not considered. Given the rapid and heterogeneous neuromuscular development that occurs during adolescence, part of the observed adaptations may have been influenced by maturational stage rather than training alone. Second, while the CT program emphasized sport-specific movements, training intensity was prescribed according to external load rather than individualized neuromuscular responses (e.g., movement velocity or potentiation effects), which may have resulted in variable training effectiveness across participants. Third, although the on-court COD test was designed to replicate badminton-specific movement demands, it did not account for perceptual or decision-making components inherent to real match play. Future research integrating perceptual-cognitive assessments is therefore warranted to better reflect the sport-specific agility required in badminton competition. In addition, the relatively small sample size (n = 32) limits the generalizability of the findings, and the restricted 8-week intervention period does not allow conclusions regarding the long-term trajectory or retention of adaptations. Although participants were instructed to avoid strenuous activity before testing and to remain fasted for at least 2 hours, other potentially influential factors such as sleep duration and quality, hydration status, nutrition, and particularly menstrual cycle phase were neither controlled nor systematically monitored. Finally, participants’s adherence to the training program was not formally documented, which creates uncertainty about whether the training effects occurred equally across all individuals. These methodological shortcomings should be addressed in future research to improve the accuracy, reliability, and interpretability of findings.

### Practical applications

This study highlights that complex training is a highly effective and developmentally appropriate approach for adolescent female badminton players. The marked responsiveness observed in this group suggests that CT protocols can leverage their heightened neuromuscular plasticity to enhance performance outcomes. Coaches are encouraged to incorporate bilateral and unilateral resistance exercises combined with multidirectional plyometric drills, with particular focus on asymmetrical movement patterns reflective of badminton demands. Moreover, given the variability in maturation and potential neuromuscular instability during adolescence, individual monitoring and appropriate adjustments to training loads are recommended to maximize benefits and support injury prevention.

## Conclusion

In conclusion, an 8-week complex training program elicited significantly greater improvements in lower limb explosive power, sprint acceleration, and multidirectional agility compared to traditional resistance training in adolescent female badminton players. These findings underscore the utility of CT as a sport-specific, developmentally appropriate intervention that supports both performance enhancement and movement quality in young female athletes. Future research should explore long-term adaptations and include neuromuscular profiling to further tailor training approaches in this unique population.

## Data Availability

The original contributions presented in the study are included in the article/supplementary material, further inquiries can be directed to the corresponding author.
